# Keratoconus: an introduction

**Published:** 2024-10-02

**Authors:** Rashmi Deshmukh, Daniel Gore, Nick Astbury

**Affiliations:** 1 Consultant: LV Prasad Eye Institute, Hyderabad, India.; 2 Consultant Ophthalmic Surgeon: Director of Refractive Surgery, Moorfields Eye Hospital, UK; 3 Honorary Clinical Lecturer, University College London, UK.; 4 Honorary Associate Professor: International Centre for Eye Health, London School of Hygiene & Tropical Medicine, London, UK.

**Knowing how to identify and refer people with keratoconus is an important first step in reducing the risk of visual impairment from this progressive condition.**

Keratoconus is a condition characterised by progressive thinning and weakening of the cornea, leading to a cone-shaped appearance that distorts vision, usually in both eyes. It typically develops in young people around the age of puberty and is more common in boys and men. Keratoconus is very rare in some countries, and common in others. In Iran, for example, more than 3 out of every hundred people have the condition; in Russia, only 2 out of every million people are known to be affected.

## Risk factors

Although the exact cause of keratoconus is not known, there are multiple risk factors associated with it, including chronic eye inflammation, eye rubbing, a family history of keratoconus, and other genetic factors such as Down syndrome, connective tissue disorders, and Leber congenital amaurosis.

## Presentation: symptoms and signs

The first signs of keratoconus are likely to be a slight blurring of vision, often in a person who is **atopic** (prone to allergies and eczema) and may excessively rub their eyes (see panel).

Other symptoms include blurred or distorted vision, often needing new spectacles (due to irregular astigmatism), difficulty seeing at night, sensitivity to light, and glare (seeing haloes around lights).

A visit to an **optician** or **optometrist** may reveal early changes that can be managed with glasses or contact lenses.

**Atopy and eye rubbing**

People with atopy, especially children and young people, are at greater risk of keratoconus. They may have **eczema** (**atopic dermatitis**), which can cause painful itching of the eyelids and surrounding skin. In people with eczema, the itching is worse when their allergies and eczema are not well managed, particularly when they are stressed, hot or exposed to an allergen such as nuts or pollen. They may also suffer from **eye allergies** (see page XX), which cause chronic eye inflammation and itching of the eyes.

People often rub their eyes in response to itching, as it can provide temporary relief. However, this usually makes the itching worse – leading to even more vigorous eye rubbing.

Children are more likely to rub their eyes, as they may not understand the risks of eye rubbing or may not be able to stop or control themselves.

**What can patients and their caregivers do?**

- To **reduce the severity of the itching over time**, seek treatment for the allergies and eczema, and adhere to any prescribed medication.
- For i**mmediate relief**, apply a **cool compress** to the eyes to relieve itching and inflammation. To make a cool compress, soak a clean cloth in water, wring it out, and place it over closed eyes. Repeat as needed.

**Top tip for physicians:** If a patient with atopy complains of blurred vision, ask whether they rub their eyes. If they say yes, this could indicate the onset of keratoconus. Refer them for an eye examination.

“Although the exact cause of keratoconus is not known, there are multiple risk factors associated with it.”

**Figure 1:**
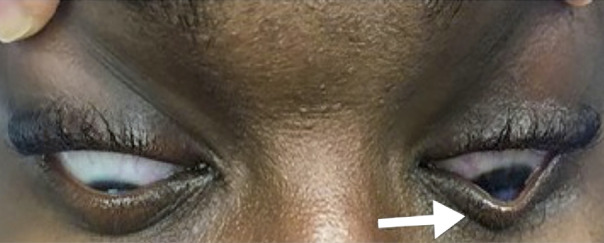
The conical shape of the cornea, outlined by the lower eyelid, is known as Munson’s sign – a sign of severe keratoconus.

In addition to changes in refraction, more severe keratoconus may be visible when the patient looks down and the conical shape of the cornea is outlined by the lower eyelid, as shown in Figure 1. This is known as Munson’s sign. These patients need specialist treatment.

© GOLD ET AL (CC BY 4.0)

## If you have a slit lamp

**Figure 2:**
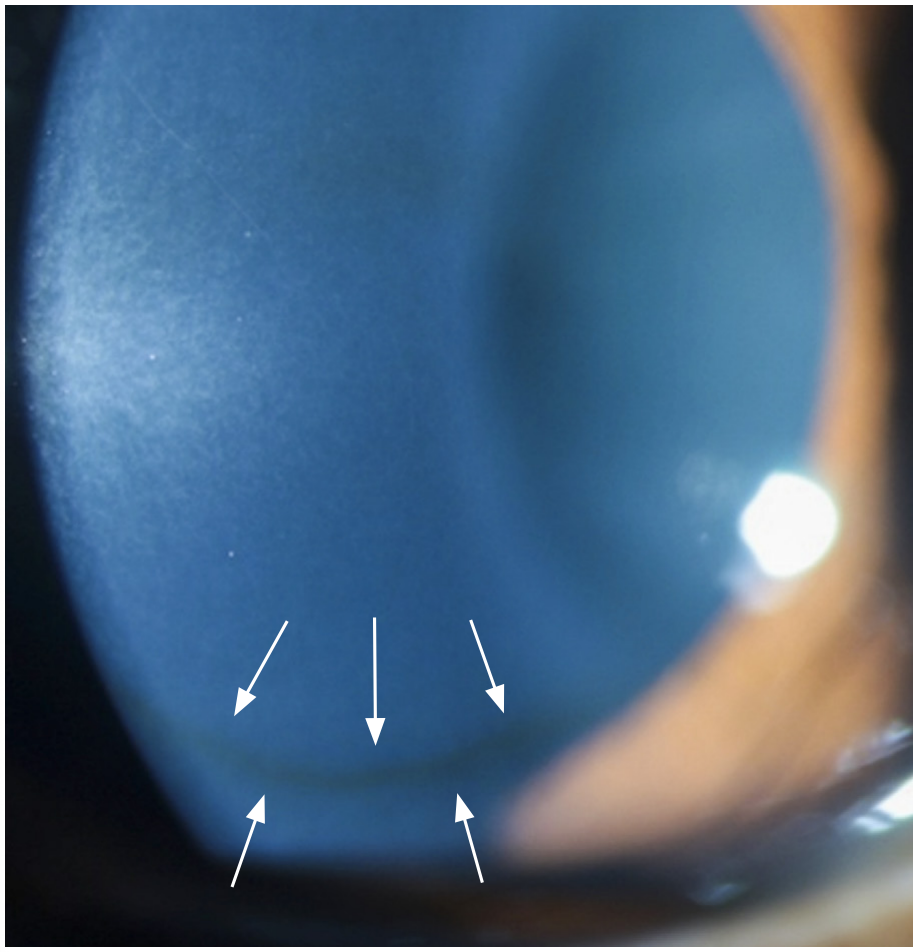
Slit lamp image showing an eye with Fleischer’s ring: a curved, light-brown line caused by iron deposits in the cornea.

**Figure 3:**
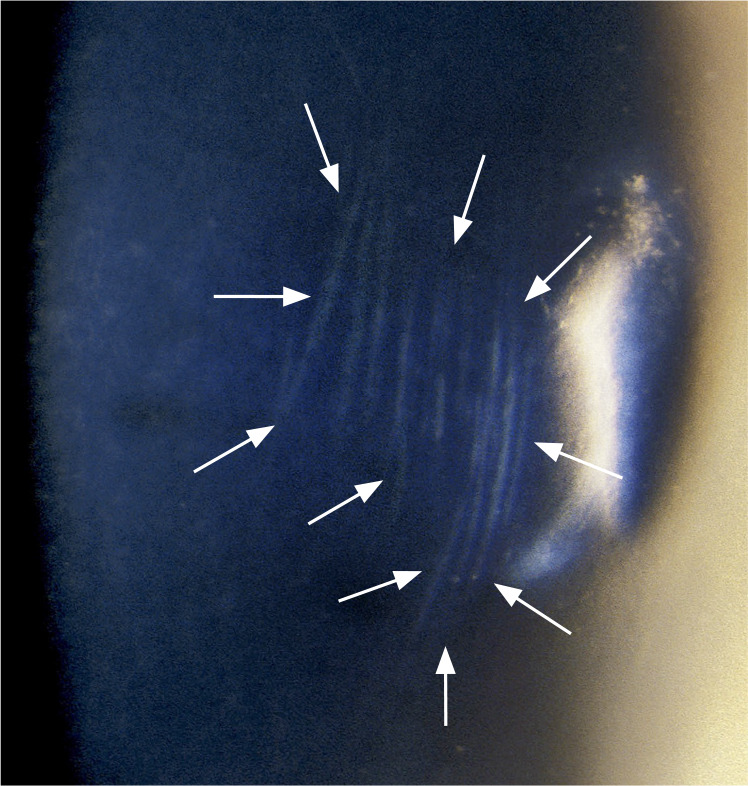
Slit lamp image showing Vogt’s striaie: faint whitish lines in the deep corneal stroma and Descemet’s membrane (see arrows).

In patients with moderate to severe keratoconus, typical slit lamp signs include corneal steepening and thinning, iron deposits in the cornea (Fleischer’s ring, Figure 2), and whitish lines in the deep corneal stroma and Descemet’s membrane (Vogt’s striae, Figure 3). As the condition progresses, splits in Descemet’s membrane can lead to corneal oedema and a whitish spot at the apex of the cone (hydrops) with subsequent poor vision and pain.

© RASHMI DESHMUKH CC BY-NC-SA 4.0

© STEVE TUFT CC BY-NC-SA 4.0

Keratoconus is often progressive, but the condition can stabilise for many people when they reach the age of 30 or above.

## What to do if you suspect your patient has keratoconus

If you think a patient may have keratoconus, refer them to a hospital with an eye department so their condition can be treated. Explain to the patient that rubbing their eyes will make the problem worse – and encourage them to stop.

If they have an eye allergy or eczema affecting the skin around the eyes (see panel), getting the right treatment for these conditions will help to reduce the itchy sensations that they are trying to relieve by rubbing their eyes.

## Diagnosis and management in the eye department

In the eye department, a diagnosis of keratoconus is made by mapping the cornea to reveal areas of irregular corneal curvature and corneal thinning.

At first, keratoconus is often managed by prescribing spectacles or rigid (gas permeable) contact lenses to improve vision. When these are no longer adequate, corneal transplant surgery may be considered.

However, an approach known as corneal cross-linking, when available, is very effective at halting progression of the condition. It involves the use of UV light after the corneal epithelium has been removed and riboflavin drops applied. This causes the corneal surface to stiffen, slowing down further thinning of the cornea and worsening of the keratoconus.

For more on diagnosis and treatment, including cross-linking, please see the next article in this issue.

